# The Smart Aging Platform for Assessing Early Phases of Cognitive Impairment in Patients With Neurodegenerative Diseases

**DOI:** 10.3389/fpsyg.2021.635410

**Published:** 2021-03-15

**Authors:** Sara Bottiroli, Sara Bernini, Elena Cavallini, Elena Sinforiani, Chiara Zucchella, Stefania Pazzi, Paolo Cristiani, Tomaso Vecchi, Daniela Tost, Giorgio Sandrini, Cristina Tassorelli

**Affiliations:** ^1^Faculty of Law, Giustino Fortunato University, Benevento, Italy; ^2^National Neurological Institute C. Mondino Foundation, Pavia, Italy; ^3^Department of Brain and Behavioral Sciences, University of Pavia, Pavia, Italy; ^4^Neurology Unit, Department of Neurosciences, Verona University Hospital, Verona, Italy; ^5^Consorzio di Bioingegneria Medica e Informatica CBIM, Pavia, Italy; ^6^Computer Graphics Division Research Centre for Biomedical Engineering (CREB), Universitat Politecnica de Catalunya, Barcelona, Spain

**Keywords:** virtual reality, serious games, cognitive impairment, global cognitive functions, neurodegenerative disease

## Abstract

**Background:** Smart Aging is a serious game (SG) platform that generates a 3D virtual reality environment in which users perform a set of screening tasks designed to allow evaluation of global cognition. Each task replicates activities of daily living performed in a familiar environment. The main goal of the present study was to ascertain whether Smart Aging could differentiate between different types and levels of cognitive impairment in patients with neurodegenerative disease.

**Methods:** Ninety-one subjects (mean age = 70.29 ± 7.70 years)—healthy older adults (HCs, *n* = 23), patients with single-domain amnesic mild cognitive impairment (aMCI, *n* = 23), patients with single-domain executive Parkinson's disease MCI (PD-MCI, *n* = 20), and patients with mild Alzheimer's disease (mild AD, *n* = 25)—were enrolled in the study. All participants underwent cognitive evaluations performed using both traditional neuropsychological assessment tools, including the Mini-Mental State Examination (MMSE), Montreal Overall Cognitive Assessment (MoCA), and the Smart Aging platform. We analyzed global scores on Smart Aging indices (i.e., accuracy, time, distance) as well as the Smart Aging total score, looking for differences between the four groups.

**Results:** The findings revealed significant between-group differences in all the Smart Aging indices: accuracy (*p* < 0.001), time (*p* < 0.001), distance (*p* < 0.001), and total Smart Aging score (*p* < 0.001). The HCs outperformed the mild AD, aMCI, and PD-MCI patients in terms of accuracy, time, distance, and Smart Aging total score. In addition, the mild AD group was outperformed both by the HCs and by the aMCI and PD-MCI patients on accuracy and distance. No significant differences were found between aMCI and PD-MCI patients. Finally, the Smart Aging scores significantly correlated with the results of the neuropsychological assessments used.

**Conclusion:** These findings, although preliminary due to the small sample size, suggest the validity of Smart Aging as a screening tool for the detection of cognitive impairment in patients with neurodegenerative diseases.

## Introduction

A growing interest in the development of accessible and easily administered neuropsychological screening tools for detecting cognitive impairment in aging, also driven by the technological advances of recent years, has resulted in excellent opportunities for improving neuropsychological evaluation in clinical practice. In this setting, virtual reality (VR) gaming and interactive video gaming have emerged as promising new ways of assessing cognitive mechanisms in a more ecological manner (e.g., Christiansen et al., 1998; Rizzo et al., 1998; Davies et al., 1999; Riva et al., 1999; Rose et al., 1999; Jack et al., 2001; Zhang et al., 2001; Kang et al., 2008; Zucchella et al., 2014a; Fabbri et al., 2019; Realdon et al., 2019). In particular, serious games (SGs), which can be defined as innovative computer games designed for purposes other than leisure (Charsky, 2010), constitute a young VR gaming subfield. These games can vary greatly in structure, but most of the ones used in neuropsychological assessment involve the generation of realistic 3D scenarios that simulate the demands of daily life and, therefore, have greater ecological validity than traditional cognitive assessments. SGs can also be self-administered (possibly after minimal training); furthermore, they provide a pleasant experience and reduce the psychological stress that can be caused by traditional screening tools (Ismail et al., 2010). Finally, being computer-based assessments, they can allow better standardization of both administration and data collection (Parsons, 2014). All these aspects are particularly useful in the diagnosis of early cognitive impairments. SGs can detect impairments in multiple cognitive domains while, thanks to the advantages outlined above, overcoming the limitations of traditional pen-and-paper tests. Therefore, they could potentially be used in place of traditional assessments to perform large-scale, low-cost screening campaigns aimed at earlier detection of cognitive impairments in aging, which in turn would allow earlier enrollment in rehabilitation programs.

As already highlighted in the literature, SGs have been successfully used for assessment purposes both in normal aging and in clinical populations, such as mild cognitive impairment (MCI) and Alzheimer's disease (AD) cohorts. Manera et al. (2015) used a cooking pot-based SG to compare groups with MCI and AD vs. healthy controls (HCs). They found the cooking game to be sensitive to between-group differences in performance, which depended on the level of cognitive impairment. Other authors, too, have provided evidence of the validity of SG-based assessments in MCI and AD (e.g., Tarnanas et al., 2014; Valladares-Rodríguez et al., 2016; Ouellet et al., 2018). To date, however, these aspects have been little explored in the field of Parkinson's disease (PD). One of the few exceptions was a study using the Virtual Multiple Errands Test (VMET), which aims to test different aspects of executive functioning (EF) by having patients explore a virtual supermarket. The authors (Cipresso et al., 2014a) compared VMET performances with performances recorded on traditional pen-and-pencil tests in cognitively normal PD patients, PD patients with MCI (PD-MCI), and HCs. The results showed that the VMET was more sensitive than traditional EF assessments in detecting EF deficits. More recently, Serino et al. (2017) used the 360° version of the Picture Interpretation Test (PIT) to compare EF in cognitively normal PD patients and HCs, and found that it seemed able to distinguish between these two groups. Together, the aforementioned studies highlight the potential of VR environments and SGs in cognitive assessment. However, more research is necessary to investigate, in detail, how they might be used for cognitive assessment in pathological aging. Given the importance, from a therapeutic perspective, of early differential diagnoses, previous studies have evaluated the ability of single assessment tools to discriminate between different forms of early cognitive impairment. To date, however, only traditional pen-and-paper tests, and not SG tools, have been evaluated (e.g., Kwak et al., 2010; Yamamoto et al., 2017; Allone et al., 2018).

Smart Aging is an SG technology-based platform developed by our group for the assessment of global cognition and specific aspects of cognition, such as memory and EF, in normal aging (Pazzi et al., 2014; Tost et al., 2014, 2015). Essentially, it integrates various games that reproduce, in 3D, different everyday life tasks. In a previous work (Bottiroli et al., 2017), we compared the results of cognitive screening performed by means of Smart Aging with the scores obtained on a traditional standardized screening test, i.e., the Montreal Overall Cognitive Assessment (MoCA), in a sample of 1,086 healthy older adults stratified by MoCA score. We found significant between-group differences in each Smart Aging task, and thus demonstrated the validity of this platform as a screening tool for cognitive functioning in normal aging. More recently, Smart Aging (Cabinio et al., 2020a) was tested for its ability to identify individuals with amnesic MCI vs. HCs, and the overall score derived from this platform (i.e., the Smart Aging total score) performed comparably, in this regard, to traditional neuropsychological tests (i.e., MoCA, Free and Cued Selective Reminding Test, Trail Making Test). In addition, Smart Aging has been shown (Zucchella et al., 2014b) to be easily administrable, even in patients unfamiliar with computerized tests. This may be explained by the fact that movements in its VR environments are performed by means of a touch screen monitor, which is easier and more intuitive to use than a mouse, even for individuals with some cognitive impairment (Cernich et al., 2007). It is, in fact, important to limit as much as possible any influence of manual skills on test results. Hence, on the basis of our previous experience, we argue that Smart Aging may complement the traditional assessment of cognitive function, and indeed serve to broaden access to neuropsychological testing.

In the present study, we set out to establish whether Smart Aging can differentiate between different types and levels of cognitive impairment in patients with neurodegenerative diseases, and whether it might therefore be used as a screening tool in these patients. Our ultimate intention is the development of an SG-technology-based assessment tool for the evaluation of cognition as a whole in an ecological context. Pathological aging can present in many different forms, and it is important to develop screening tools able to distinguish between them and, therefore, able to identify factors that may affect a patient's disease course and increase opportunities for interventions designed to delay or prevent progression to dementia. In the present study, we tested the Smart Aging platform in patients with different types of MCI (single-domain amnesic MCI—aMCI—and single-domain executive MCI—PD-MCI) and in patients with mild AD. A sample of healthy older adults was included as the control group. We expected that patients with different cognitive profiles would show different Smart Aging performance trends. Performances across groups were evaluated in terms of accuracy, time spent performing tasks, and distance covered within the virtual environment. We also considered the Smart Aging total score (obtained from the difference between accuracy, time, and distance), which could represent a final index of performance and reflect global functioning. Giving that SGs use automated systems for scoring performances (Clauser et al., 2002), it might therefore capture the complexity of cognitive functioning in everyday situations, better than traditional assessments do (Fortin et al., 2003). In particular, evaluation of indices such as time and distance, in addition to accuracy, may better reveal whether individuals are able to use skills and strategies effectively in order to facilitate their responses to environmental demands. Finally, we also evaluated associations between Smart Aging scores—i.e., the global scores recorded for three indices (accuracy, time, and distance) and the Smart Aging total score—and performances on traditional neuropsychological tests. Given that this platform was expected to reflect global cognitive functioning, correlations were first carried out with traditional screening tests (i.e., MMSE and MoCA), and then with measures of specific cognitive functions.

## Materials and Methods

### Design of the Comparative Study

This study was designed to compare cognitive performance in normal aging and early cognitive impairment using the Smart Aging platform. To this end, we evaluated four groups of subjects: aMCI, PD-MCI, and mild AD patients, and a group of HCs.

### Participants

A total sample of 91 subjects (mean age = 70.29 ± 7.70 years) took part in this study. It comprised patients diagnosed with aMCI (*n* = 23), mild AD (*n* = 25), and PD-MCI (*n* = 20), who were recruited and enrolled from the Neuropsychology/Alzheimer's Disease Assessment Unit and Neurorehabilitation Unit of the IRCCS Mondino Foundation.

The inclusion criteria were:

- a diagnosis of mild AD, aMCI, or PD-MCI according to widely accepted diagnostic criteria (McKhann et al., 2011, for mild AD; Albert et al., 2011, for aMCI, and Litvan et al., 2012, for PD-MCI);
- a Mini-Mental State Examination (MMSE) score > 20 in patients with mild AD;
- age between 60 and 85 years;
- educational level ≥5 years.
- The exclusion criteria were:
- other causes of cognitive impairment due to preexisting conditions (e.g., aphasia, neglect);
- concomitant severe psychiatric diseases or other neurological conditions (e.g., depression and behavioral disorders);
- severe sensory or motor disturbances liable to interfere with the assessment;
- deep brain stimulation.

A group of age-, gender-, and education-matched community-dwelling healthy older adults (HCs, *n* = 23) was also included. HCs were recruited among patients' caregivers. They were native Italian speakers and received no tangible incentive to participate.

Written informed consent was obtained from all the participants; the consent document and study protocol had local ethics committee approval. Participant characteristics are reported in Table 1.

**Table 1:** Demographic characteristics and traditional neuropsychological assessment scores of study participants.

	**HCs (*n* = 23)**	**Mild AD (*n* = 25)**	**aMCI (*n* = 23)**	**PD-MCI (*n* = 20)**	***p***
Age	69.43 (6.83)	73.52 (6.86)	69.74 (9.27)	67.85 (6.84)	0.08
Gender (F)	12 (52%)	13 (52%)	12 (52%)	9 (45%)	0.96
Education (years)	10.39 (2.55)	8.84 (4.36)	10.44 (4.64)	9.20 (3.61)	0.38
MMSE	26.99 (2.58)^*^^+^	22.79 (2.05)^*^^°^^#^	25.09 (2.78)^°^^+^	25.44 (2.16)^#^	<0.001
MoCA	26.60 (3.47)^*^^+^^z^	17.84 (2.94)^*^	19.08 (2.73) ^+^	16.74 (2.43)^z^	<0.001
**Fluency^§^**						
Phonological	−0.11 (0.58)^*^^+^^z^	−0.67 (0.65)^*^	−0.68 (0.73)^+^	−0.89 (0.77)^z^	0.002
Semantic	−0.15 (0.92)^*^^+^^z^	−1.78 (0.90)^*^	−1.29 (0.72) ^+^	−1.63 (0.55)^z^	<0.001
**TMT^§^**						
Part A	0.31 (2.84)^*^^z^	2.81 (2.26)^*^^°^	1.18 (1.54)^°^	2.13 (1.49)^z^	0.001
Part B	−0.12 (1.06)^*^^z^	2.41 (2.09)^*^	1.15 (1.60)	1.93 (2.05)^z^	<0.001
**FCSRT^§^**						
Immediate free recall	−0.12 (1.17)^*^^+^^z^	−3.08 (0.97)^*^^°^^#^	−1.93 (1.32)^°^^+^	−1.74 (1.37)^#^^z^	<0.001
Immediate total recall	−0.94 (1.29)^*^^+^	−3.75 (2.52)^*^^#^	−2.20 (2.11)^+^	−1.67 (2.85)^#^	<0.001
Delayed free recall	−1.00 (1.16)^*^	−3.07 (1.13)^*^^°^^#^	−1.43 (2.97)^°^	−1.42 (1.27)^#^	<0.001
Delayed total recall	−0.16 (1.42)^*^^+^	−3.20 (2.95)^*^^#^	−2.45 (2.38)^+^	−1.17 (2.10)^#^	<0.001

* Significant differences between HC and mild AD.

+ *Significant differences between HC and aMCI*.

z *Significant differences between HC and PD-MCI*.

° *Significant differences between mild AD and aMCI*.

# *Significant differences between mild AD and PD-MCI. TMT = Trial Making Test; FCSRT = Free and Cued Selective Reminding Test*.

§ *, Z scores*.

### Traditional Neuropsychological Assessment

In all cases, before the participants performed the Smart Aging test, their global cognitive functioning was assessed using the following traditional cognitive screening tests: MMSE (Magni et al., 1996) and MoCA (Conti et al., 2015).

Participants were also administered a neuropsychological battery including (a) phonological (Carlesimo et al., 1996) and semantic fluency (Novelli et al., 1986) tests, to assess logical-executive functions and language; (b) the Trail Making Test (TMT, parts A and B) (Giovagnoli et al., 1996), to assess executive functions, mental flexibility, visual search ability, and processing speed; and (c) the Free and Cued Selective Reminding Test (FCSRT) (Frasson et al., 2011), focusing on immediate and delayed free and total recall, to evaluate encoding and retrieval phases of the memorization processes.

### The Smart Aging Platform

**Figure 1:**
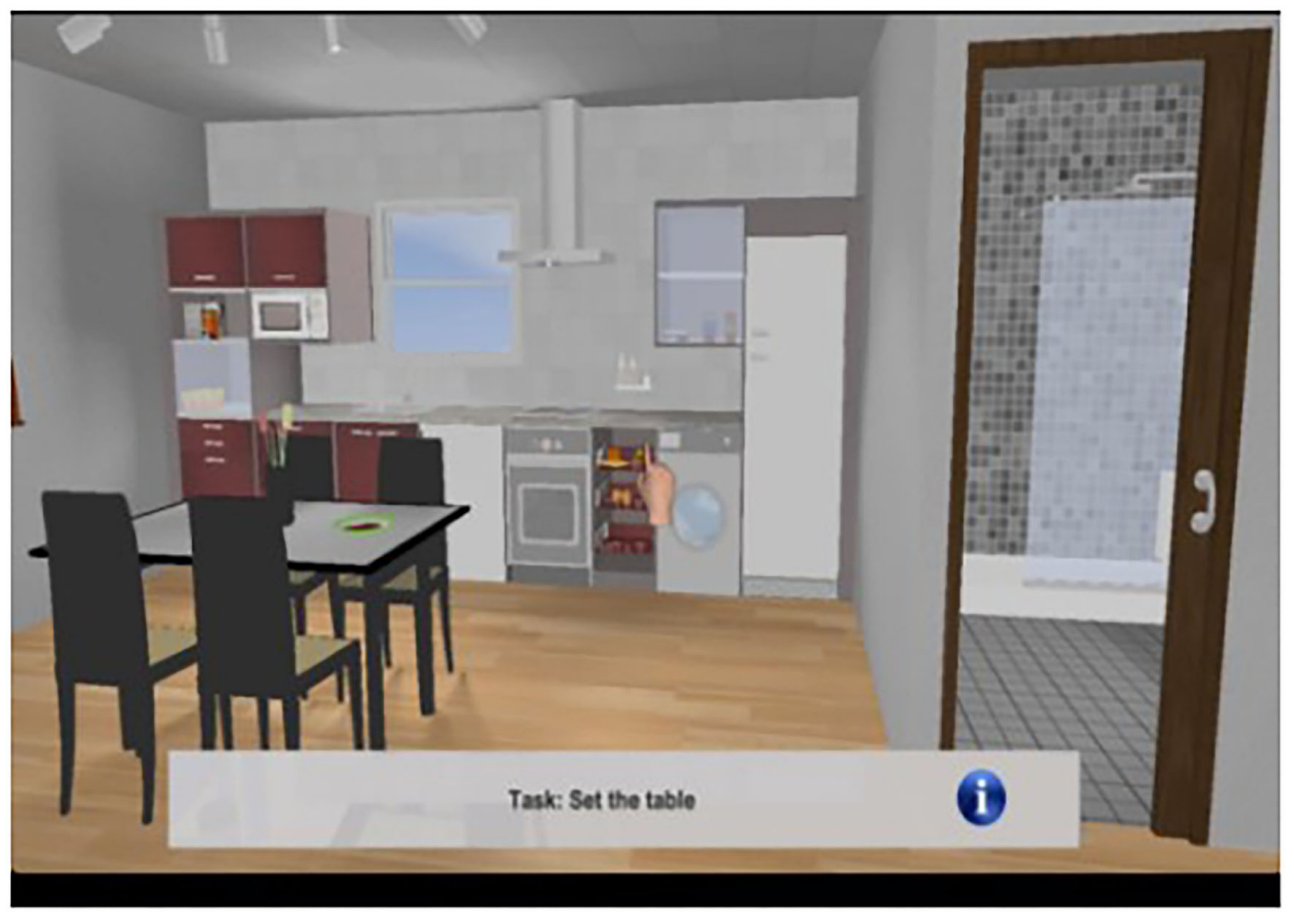
An example of the virtual scenarios used in the Smart Aging platform.

As described elsewhere (Pazzi et al., 2014; Tost et al., 2014, 2015; Zucchella et al., 2014b; Bottiroli et al., 2017), Smart Aging is an SG platform based on a first-person paradigm and administered in the presence of a neuropsychologist. The virtual 3D environment is a loft apartment that brings together, in a small space, the basic elements of the environmental interactions that occur in the setting of a private home: a kitchen corner, a bedroom corner, and a living room corner (see Figure 1). Participants use a touch screen monitor to navigate and interact with the environment. The Smart Aging platform has been designed to engage participants in task-specific scenarios where they perform five tasks, related to everyday life activities, that evaluate several cognitive functions (e.g., EF, attention, memory, and visuo-spatial orientation) (see Table 2 for a description of the tasks). Execution of the whole game takes from 10 to 30 min. As the participant experiences the virtual environment and performs the tasks, the system records various data (positions, times, and actions). The scores provide a picture of the participant's cognitive functions. In particular, the system computes separate sets of indices for each task. For four of the five tasks, we considered accuracy, time, and distance; for task 4, a 2D task not entailing navigation in the environment, we considered only accuracy and time. Accuracy was measured as the total number of correct actions while completing each of the tasks. In particular, for tasks 1, 4, and 5, it referred to the total number of objects correctly remembered, whereas for tasks 2 and 3, it corresponded to the total number of correct actions performed while completing each of these tasks. For task 3, we also considered correct recall of the telephone number needed to make the phone call, as well as performance of the prospective memory action, i.e., remembering to switch on the TV at the end of the task. Time, on the other hand, referred to the time taken to accomplish each task, from start to finish. Distance was the number of meters covered in the loft while performing each task, from start to finish. More information is available in Bottiroli et al. (2017).

**Table 2:** The Smart Aging tasks.

**Task**	**Picture**
**Task 1—Object search** After exploring the kitchen, the subject is asked to look for a list of objects.	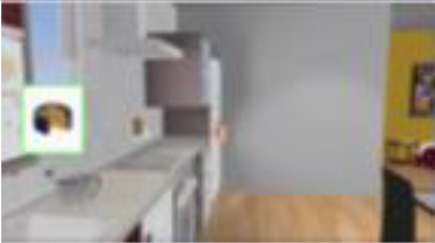
**Task 2—Water flowers while listening to the radio** The subject is asked to turn on the radio and press the spacebar every time the word “sun” is aired, while watering the flowers on the windowsill in the dining room.	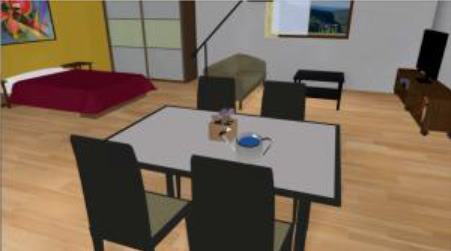
**Task 3—Make a phone call** The person is asked to make a phone call using the phone book and the phone placed on the bedside table. The subject is asked to remember to turn the TV on after dialing the number.	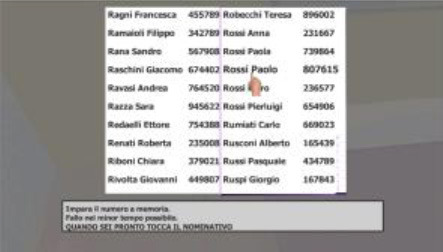
**Task 4—Choose the right object** A 2D screen with 24 images of objects is shown. The subjects has to identify the 12 objects presented in task 1.	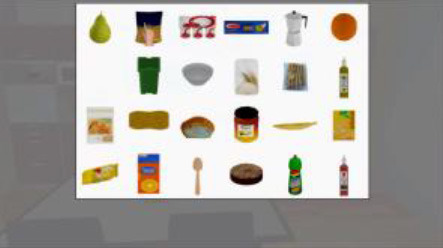
**Task 5—Find the objects** The subject is positioned in front of the kitchen, and he/she is asked to find each of the objects that he looked for in task 1.	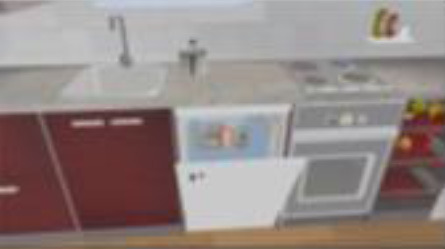

### Statistical Analysis

In accordance with previous research (Bottiroli et al., 2017), for each Smart Aging task, we considered accuracy, time, and distance, which were converted into *z*-score units. We then computed a global score per index, in each case obtained as the sum of the scores recorded over the five tasks. Finally, we computed the Smart Aging total score, obtained by calculating the sum of (or difference between, in the case of reverse scores, i.e., time and distance) the scores of all five tasks. We used univariate analysis of variance (ANOVA) in order to compare normally distributed variables between groups. The Tukey *post-hoc* test with 0.05 level of significance was applied to evaluate between-group differences. As the distribution of the Smart Aging data was not normal, group comparisons were performed using non-parametric Kruskal–Wallis tests followed by Mann–Whitney *U*-tests corrected for multiple comparisons. A series of receiver operating characteristic (ROC) analyses was performed to evaluate the relationship between sensitivity and specificity of the global accuracy, time, and distance scores on each of the five tasks, and of the Smart Aging total score, for identifying the four groups. The area under the ROC curve (AUC) gives the proportion of cases that are correctly discriminated by the considered variables. To this end, we compared each group with the other three (i.e., HCs vs. mild AD + aMCI + PD-MCI; mild AD vs. HCs + aMCI + PD-MCI; aMCI vs. HCs + mild AD + PD-MCI; and PD-MCI vs. HCs + mild AD + aMCI). For the Smart Aging total score, we also performed the ROC analysis comparing HCs vs. mild-AD alone, and mild AD vs. aMCI and PD-MCI separately, in order to avoid biases related to the differences between the clinical entities considered. This analysis was restricted to the Smart Aging total score as this was expected to be indicative of the presence/absence of cognitive impairment. Effect sizes were calculated by using G^*^Power 3 (Faul et al., 2007). Finally, Pearson's correlations were used to detect associations between Smart Aging scores and neuropsychological tests. These analyses were carried out first on MMSE and MoCA, as these are our gold standard traditional screening tests, and then using the rest of the neuropsychological battery. We set the significance level alpha at 0.05 for parametric tests, while a value of 0.0125 (0.05/4) was applied for non-parametric tests involving the four groups. The SPSS 23.0 statistical software package was used to perform all the statistical analyses.

## Results

### Participant Characteristics

The four groups were similar (Table 1) in terms of age, *F*_(3,90)_ = 2.37; *p* = 0.08, and years of education, *F*_(3,90)_ = 1.03; *p* = 0.38. The proportion of female and male participants was similar across the groups, χ^2^_(3)_ = 0.32; *p* = 0.96.

### Traditional Neuropsychological Evaluations

MMSE scores differed significantly between the four groups, *F*_(3,90)_ = 12.46, *p* < 0.001 (Table 1). Specifically, the score was lower in the mild AD group than in the other three groups, while the aMCI group scored lower than the HCs. No other comparisons of MMSE scores showed differences. Significant differences between groups were also found in the MoCA scores, *F*_(3,90)_ = 52.19, *p* < 0.001. In this case, the HCs outperformed the three other groups, which all performed similarly to each other.

The HCs recorded significantly higher scores than the three other groups both on phonological and on semantic fluency tests, *F*_(3,87)_ = 5.42, *p* = 0.002 and *F*_(3,87)_ = 19.78, *p* < 0.001, respectively, whereas the three patient groups performed similarly to each other.

On the TMT part A, *F*_(3,86)_ = 6.16, *p* = 0.001, the mild AD patients were outperformed by the HCs and the aMCI group, while the HCs outperformed the PD-MCI group. No other significant between-group differences were found. On the TMT part B, *F*_(3,68)_ = 8.35, *p* < 0.001, the HCs outperformed both the mild AD and the PD-MCI patients, but no other significant differences emerged between the groups.

On FCSRT immediate free recall, *F*_(3,86)_ = 23.75, *p* < 0.001, the HCs outperformed the three patient groups, and the mild AD patients were outperformed by the aMCI and PD-MCI groups, which performed similarly to each other. On FCSRT immediate total recall, *F*_(3,86)_ = 10.56, *p* < 0.001, HCs outperformed the mild AD and aMCI groups; the mild AD patients were outperformed by the PD-MCI group. The other groups performed similarly to each other. On FCSRT delayed free recall, *F*_(3,86)_ = 10.57, *p* < 0.001, the mild AD group was outperformed by the other three groups, which all performed similarly to each other. Finally, on FCSRT delayed total recall, *F*_(3,85)_ = 7.99, *p* < 0.001, the HCs outperformed the mild AD and aMCI patients, and the mild AD group was also outperformed by the PD-MCI patients. No other between-group differences were found.

### Smart Aging Results

The means and standard deviations for accuracy, time, and distance (expressed in *z* scores) are reported in Table 3 and the corresponding analyses in Table 4.

**Table 3:** Z scores—means and (standard deviations)—in the Smart Aging tasks as a function of group.

**Smart Aging tasks**	**HCs (*n* = 23)**	**Mild AD (*n* = 25)**	**aMCI (*n* = 23)**	**PD-MCI (*n* = 20)**	***p***
**Accuracy**					
Task 1	−0.02^*^^+^^z^ (1.06)	−2.67^*^ (0.86)	−2.16^+^ (1.04)	−2.46^z^ (0.93)	<0.001
Task 2	0.11 (1.31)	−0.47 (1.54)	0.00 (1.91)	0.33 (1.48)	0.15
Task 3	−0.07^*^^+^^z^ (1.78)	−1.86^*^ (1.93)	−1.07^+^ (0.99)	0.33^z^ (1.48)	<0.001
Task 4	−0.12^*^^+^^z^ (1.32)	−2.74^*^^°^^#^ (1.21)	−1.53^+^^°^ (1.58)	−1.05^#^^z^ (1.07)	<0.001
Task 5	0.03^*^^+^^z^ (0.78)	−3.44^*^^#^ (1.07)	−2.68^+^ (1.25)	−2.46^#^^z^ (1.31)	<0.001
Global accuracy score	−0.26^*^^+^^z^ (4.41)	−11.17^*^^°^^#^ (4.14)	−7.44^+^^°^ (4.23)	−6.83^#^^z^ (3.23)	<0.001
**Time**					
Task 1	0.51^*^^+^^z^ (1.29)	2.15^*^ (0.88)	1.60^+^ (0.80)	2.18^z^ (1.20)	<0.001
Task 2	0.20^*^^+^^z^ (1.12)	1.86^*^ (0.67)	1.92^+^ (1.69)	1.40 ^z^ (1.95)	<0.001
Task 3	−0.28^*^^+^^z^ (0.78)	2.71^*^^°^ (1.57)	1.71^+^^°^ (0.91)	1.71^z^ (1.54)	<0.001
Task 4	0.05 (0.82)	0.24 (0.97)	0.70 (2.12)	0.22 (1.39)	0.57
Task 5	0.01^*^^+^^z^ (1.00)	1.92^*^ (0.84)	1.94^+^ (0.74)	1.88^z^ (0.98)	<0.001
Global time score	0.50^*^^+^^z^ (3.26)	8.65^*^ (2.37)	7.17^+^ (2.42)	6.61^z^ (4.47)	<0.001
**Distance**					
Task 1	−0.09^*^^+^ (1.01)	−1.99^*^^°^^#^ (1.08)	−1.05^+^^°^ (1.52)	−0.18^#^ (1.92)	<0.001
Task 2	−0.06^*^^z^ (0.89)	−1.27^*^^°^ (0.56)	−0.34^°^ (1.69)	−0.98^z^ (0.74)	<0.001
Task 3	−0.02^*^^+^^z^ (0.56)	−2.32^*^^°^ (0.99)	−1.27^+^^°^ (2.43)	−2.26^z^ (1.09)	<0.001
Task 5	−0.01^*^ (1.11)	−0.86^*^^°^^#^ (0.90)	−0.24^°^ (1.18)	0.54^#^ (1.86)	0.01
Global distance score	0.17^*^^+^^z^ (2.27)	−5.19^*^^°^^#^ (2.39)	−1.30^+^^°^ (5.70)	−1.11^#^^z^ (3.40)	<0.001
**Smart Aging total score**	1.22^*^^+^^z^ (4.14)	−15.23^*^ (5.86)	−15.18^+^ (6.43)	−13.19^z^ (6.55)	<0.001

Task 1 = Object search; Task 2 = Water flowers while listening to the radio; Task 3 = Make a phone call; Task 4 = Choose the right object; Task 5 = Find the objects. Distance is not reported for Task 4 because it is a 2D task not involving navigation.

* Significant differences between HCs and mild AD.

+ Significant differences between HCs and aMCI.

z Significant differences between HCs and PD-MCI.

° Significant differences between mild AD and aMCI.

# *Significant differences between mild AD and PD-MCI*.

**Table 4:** Between-group comparisons of Smart Aging task performances using the Kruskal–Wallis test and then the Mann–Whitney test for significant differences.

**Smart Aging tasks**	**Kruskal–Wallis test**			**HC vs. Mild AD**		**HC vs. aMCI**		**HC vs. PD-MCI**		**Mild AD vs. aMCI**		**Mild AD vs. PD-MCI**	
	**χ^**2**^**	**df**	***p***	***U***	***p***	***U***	***p***	***U***	***p***	***U***	***p***	***U***	***p***
**Accuracy**													
Task 1	37.34	3	<0.001	41.50	<0.001	57.50	<0.001	35.50	<0.001	–	–	–	–
Task 2	5.38	3	0.15	–	–	–	–	–	–	–	–	–	–
Task 3	34.70	3	<0.001	59.50	<0.001	77.00	<0.001	55.00	<0.001	–	–	–	–
Task 4	35.41	3	<0.001	51.00	<0.001	106.50	<0.001	90.00	0.001	159.00	0.008	76.50	<0.001
Task 5	49.41	3	<0.001	8.00	<0.001	20.00	<0.001	19.50	<0.001	–	–	151.0	0.022
Global accuracy score	44.43	3	<0.001	29.00	<0.001	51.00	<0.001	49.00	<0.001	152.50	0.005	95.00	<0.001
**Time**													
Task 1	29.87	3	<0.001	63.00	<0.001	94.00	<0.001	49.00	<0.001	–	–	–	–
Task 2	39.40	3	<0.001	33.50	<0.001	49.40	<0.001	67.00	<0.001	–	–	–	–
Task 3	46.42	3	<0.001	10.00	<0.001	28.00	<0.001	34.00	<0.001	161.00	0.009	–	–
Task 4	2.00	3	0.57	–	–	–	–	–	–	–	–	–	–
Task 5	25.20	3	<0.001	77.00	<0.001	71.00	<0.001	59.00	<0.001	–	–	–	–
Global time score	39.70	3	<0.001	24.00	<0.001	27.00	<0.001	49.00	<0.001	–	–	–	–
**Distance**													
Task 1	24.55	3	<0.001	50.00	<0.001	138.00	<0.015	–	–	171.50	0.016	90.50	0.001
Task 2	22.32	3	<0.001	46.00	<0.001	–	–	71.00	0.001	193.00	0.034	–	–
Task 3	32.75	3	<0.001	39.00	<0.001	85.00	<0.001	41.50	<0.001	202.00	0.032	–	–
Task 5	11.43	3	<0.001	141.00	0.007	–	–	–	–	178.00	0.022	112.00	0.005
Global distance score	39.79	3	<0.001	20.00	<0.001	27.00	<0.001	119.00	0.049	176.00	0.022	69.00	<0.001
**Smart Aging total score**	44.58	3	<0.001	7.00	<0.001	3.00	<0.001	15.00	<0.001	–	–	–	–

*Task 1 = Object search; Task 2 = Water the flowers while listening to the radio; Task 3 = Make a phone call; Task 4 = Choose the right object; Task 5 = Find the objects. Distance is not reported for Task 4 because it is a 2D task not involving navigation. There is no column comparing the aMCI and PD-MCI patients as these two groups showed no significant differences*.

#### Accuracy

The four groups showed significant differences in accuracy scores on all the tasks (except Task 2, on which they scored similarly) and in global accuracy.

On Task 1, the HCs outperformed the mild AD (*d* = 2.74), aMCI (*d* = 2.04), and PD-MCI (*d* = 2.45) groups, which all performed similarly (*p* > 0.09).

On Task 3, the HCs recorded higher scores than the mild AD (*d* = 0.96), aMCI (*d* = 0.69), and PD-MCI (*d* = 0.24) patients, with no differences found between the three clinical groups (*p* > 0.09).

On Task 4, too, the HCs outperformed the mild AD (*d* = 2.07), aMCI (*d* = 0.97), and PD-MCI (*d* = 0.77) groups. The mild AD patients scored lower than the aMCI (*d* = 0.86) and PD-MCI (*d* = 1.48) ones, which instead performed similarly to each other (*p* = 0.38).

On Task 5, the HCs again outperformed the mild AD (*d* = 3.71), aMCI (*d* = 2.60), and PD-MCI (*d* = 2.31) groups. The mild AD patients scored lower than the PD-MCI ones (*d* = 0.82). No other between-group differences were found (*p* > 0.09).

The HCs recorded a higher global accuracy score than all three clinical groups: mild AD (*d* = 2.55), aMCI (*d* = 1.67), and PD-MCI (*d* = 1.70). The mild AD patients were outperformed by the aMCI (*d* = 0.90) and PD-MCI (*d* = 1.17) groups, which performed similarly to each other (*p* = 0.63).

The ROC curve and the AUC of global accuracy scores were first measured by comparing HCs vs. mild AD + aMCI + PD-MCI patients. The AUC was 0.975 (95% confidence interval, 0.828–1.00, *p* < 0.001). When comparing mild AD vs. HCs + aMCI + PD-MCI groups, the AUC was 0.168 (95% confidence interval, 0.082–0.253, *p* < 0.001). Global accuracy was not a significant predictor of aMCI vs. HCs + mild AD + PD-MCI (AUC: 0.437–95% confidence interval, 0.315–0.559, *p* = 0.37) or for PD-MCI vs. HCs + mild AD + aMCI (AUC: 0.496–95% confidence interval, 0.377–0.614, *p* = 0.95).

#### Time

The four groups showed significant differences both in the time scores recorded on each of the tasks (except Task 4, on which they scored similarly) and in the global time score.

On Task 1, the HCs were faster than the mild AD (*d* = 1.48), aMCI (*d* = 1.01), and PD-MCI (*d* = 1.34) patients. The three clinical groups did not differ from each other (*p* > 0.10).

On Task 2, the HCs were faster than the mild AD (*d* = 1.80), aMCI (*d* = 1.20), and PD-MCI (*d* = 0.75) groups, which again performed similarly (*p* > 0.23).

On Task 3, the HCs were faster than the mild AD (*d* = 2.41), aMCI (*d* = 2.35), and PD-MCI (*d* = 1.63) groups. The mild AD patients were slower than the aMCI ones (*d* = 0.78), but no other differences were found between the groups (*p* > 0.11).

As for Task 5, the HCs were faster than the mild AD (*d* = 2.07), aMCI (*d* = 2.19), and PD-MCI (*d* = 1.89) groups, which did not differ from each other (*p* < 0.58).

As regard the global time score, the HCs were faster than mild AD (*d* = 2.86), aMCI (*d* = 2.32), and PD-MCI (*d* = 1.56) groups, which all performed similarly (*p* > 0.10).

The ROC curve and the AUC of the global time score were initially measured by comparing HCs vs. mild AD + aMCI + PD-MCI patients; the AUC was 0.937 (95% confidence interval, 0.848–1.000, *p* < 0.001). We then measured the ROC curve by comparing mild AD vs. HC + aMCI + PD-MCI patients, and the AUC was 0.250 (95% confidence interval, 0.150–0.349, *p* < 0.001). The global time score was not a significant predictor of aMCI vs. HCs + mild AD + PD-MCI (AUC: 0.405–95% confidence interval, 0.283–0.527, *p* = 0.18) or PD-MCI vs. HCs + mild AD + aMCI (AUC: 0.421–95% confidence interval, 0.287–0.556, *p* = 0.31).

#### Distance

The groups differed significantly in terms of the distance covered in each of the four tasks and also in the global distance score.

On Task 1, the mild AD patients covered less distance than the other groups: aMCI (*d* = 0.71), PD-MCI (*d* = 1.16), and HCs (*d* = 1.82); the aMCI patients covered less distance than the HCs (*d* = 0.74). No other differences were found between the groups (*p* > 0.22).

On Task 2, the mild AD patients covered less distance than the aMCI ones (*d* = 0.74) and the HCs (*d* = 1.63). The PD-MCI patients covered less distance than the HCs (*d* = 1.12). No other between-group differences were found (*p* > 0.20).

On Task 3, the mild AD patients again covered less distance than the aMCI ones (*d* = 0.56) and the HCs (*d* = 2.86). In addition, the aMCI (*d* = 0.71) and PD-MCI (*d* = 2.58) groups covered more distance than the HCs. No other between-group differences were found (*p* > 0.11).

On Task 5, the mild AD patients covered more distance than the other three groups: aMCI (*d* = 0.03), PD-MCI (*d* = 0.06), and HCs (*d* = 2.87), which all performed similarly (*p* > 0.28).

The global distance score showed that the mild AD group covered less distance than the aMCI patients (*d* = 0.89), PD-MCI patients (*d* = 1.39), and HCs (*d* = 2.30); the aMCI (*d* = 0.34) and PD-MCI (*d* = 0.44) groups covered more distance than the HCs. No other between-group differences were found (*p* = 0.21).

When comparing HCs vs. the mild AD + aMCI + PD-MCI groups, the AUC of the global distance score was 0.237 (95% confidence interval, 0.140–0.333, *p* < 0.001). When measuring the ROC curve for mild AD vs. HCs + aMCI + PD-MCI, the AUC was 0.829 (95% confidence interval, 0.743–0.914, *p* < 0.001). The global distance score was not a significant predictor of aMCI vs. HC + mild AD + PD-MCI (AUC: 0.479–95% confidence interval, 0.317–0.640, *p* = 0.76), or of PD-MCI vs. HC + mild AD + aMCI (AUC: 0.409–95% confidence interval, 0.282–0.536, *p* = 0.23).

#### Smart Aging Total Score

As for this score, the HCs outperformed the mild AD (*d* = 3.24), aMCI (*d* = 3.03), and PD-MCI (*d* = 2.63) groups. The three clinical groups did not differ from each other (*p* > 0.29).

When measuring the ROC curve of the Smart Aging total score for HCs vs. mild AD + aMCI + PD-MCI, the AUC was 0.982 (95% confidence interval, 0.959–1.000, *p* < 0.001). On comparison of mild AD vs. HCs + aMCI + PD-MCI, the AUC was found to be 0.304 (95% confidence interval, 0.192–0.417, *p* = 0.005). Comparing aMCI vs. HC + mild AD + PD-MCI gave an AUC of 0.314 (95% confidence level, 0.201–0.427, *p* = 0.009). This index was not a significant predictor of PD-MCI vs. HC + mild AD + aMCI (AUC: 0.421–95% confidence interval, 0.288–0.554, *p* = 0.30).

We then performed separate ROC analyses. For HCs vs. mild AD, the AUC was 0.986 (95% confidence interval, 0.962–1.000, *p* < 0.001). Instead, this index was not a significant predictor of mild AD vs. aMCI (AUC: 0.484–95% confidence interval, 0.315–0.652, *p* = 0.85) and mild AD vs. PD-MCI (AUC: 0.414–95% confidence interval, 0.236–0.592, *p* = 0.35).

### Correlations

As shown in Table 5, the Smart Aging global accuracy and global distance scores and the Smart Aging total score correlated positively with both MMSE and MoCA performances, whereas negative correlations were found between the global time score and MMSE and MoCA. When considering specific neuropsychological tests (fluencies, TMT, and FCSRT), the same trend was found: positive associations with the Smart Aging global accuracy, global distance and total scores, but negative associations with the global time score. The only exception was the lack of an association between phonological fluency and the global distance score.

**Table 5:** Correlations of Smart Aging global task and total scores with traditional neuropsychological test performances.

	**Accuracy**	**Time**	**Distance**	**Smart Aging total score**
MMSE	0.36^**^	−0.42^**^	0.36^**^	0.37^**^
MoCA	0.27^**^	−0.63^**^	0.26^**^	0.64^**^
Phonological fluency	0.22^*^	−0.31^**^	0.10	0.30^**^
Semantic fluency	0.59^**^	−0.49^**^	0.39^**^	0.49^**^
TMT part A	−0.49^**^	0.50^**^	−0.33^**^	−0.39^**^
TMT part B	−0.48^**^	0.53^**^	−0.33^**^	−0.41^**^
FCSRT immediate free recall	0.67^**^	−0.65^**^	0.44^**^	0.58^**^
FCSRT immediate total recall	0.53^**^	−0.51^**^	0.46^**^	0.40^**^
FCSRT delayed free recall	0.49^**^	−0.37^**^	0.24^*^	0.40^**^
FCSRT delayed total recall	0.56^**^	−0.44^**^	0.41^**^	0.40^**^

** p < 0.01;

* *p < 0.05*.

## Discussion

The main aim of the present study was to evaluate the Smart Aging platform as a potential screening tool for differentiating between patients with early neurodegenerative disease and different types and levels of cognitive impairment. To this end, we examined cognitive performances in patients with (a) single-domain amnesic MCI, (b) single-domain executive MCI (PD-MCI), and (c) mild AD, as well as in (d) healthy older adults. Using this tool, we calculated global accuracy, time, and distance scores, each calculated taking into account performances across the five Smart Aging tasks, as well as a composite total score (i.e., Smart Aging total score) calculated as the sum of (or difference between, in the case of reverse scores, i.e., time and distance) the scores recorded on each of the five tasks.

In general, the global accuracy, time and distance scores showed marked differences between the healthy older adults and the mild AD, aMCI, and PD-MCI patients, as well as between the mild AD patients and the other three study groups. We did not find differences between the aMCI and PD-MCI groups. A similar pattern was found when considering these performance indices within each of the five tasks (with the sole exceptions of accuracy on task 2 and time on task 4, in which the groups did not differ). Taken together, these findings seem to suggest that the Smart Aging platform is particularly sensitive as a means of detecting differences between the two opposite ends of the normal/impaired continuum of cognitive functioning in aging, but slightly less sensitive when it comes to distinguishing between the variants that lie along it; this was evident when considering both the global and the single task performances. The lack of between-group differences in accuracy on task 2, together with the fact that all the groups performed it well in comparison with the other four Smart Aging tasks, might indicate that it was comparatively easy. Instead, the lack of differences between the four groups in the time taken to perform task 4 could depend on the fact that this was a 2D task, and as a consequence, timing was not a crucial factor for comparing the groups. The ROC curves and AUC measurements for the performance indices considered in this study showed the platform to have good discriminative capacity in distinguishing healthy participants and mild AD patients from the other groups. Interestingly, we also found that the Smart Aging total score performed well in discriminating aMCI patients from the other three groups. The fact that no similar discriminative ability was found in a previous study using Smart Aging in normal aging participants stratified according to MoCA scores (Bottiroli et al., 2017) highlights the “true” discriminative power of this game platform when used in populations with neurodegenerative diseases.

Rather surprisingly, no differences in Smart Aging scores were found between the patients with different types of MCI, as might instead have been expected, considering that the two conditions reflect the involvement of anatomically and functionally diverse structures, with hippocampal atrophy (Evans et al., 2010) being found in aMCI, and basal ganglia degeneration (McKinlay et al., 2010) in PD-MCI. However, it is important to consider that the present study included only patients with single-domain MCI, which might be characterized by less functional impairment than multiple-domain MCI, as already suggested by others (Aretouli and Brandt, 2010). Future studies, also considering MCI patients with other subtypes of impairment, are needed to better clarify this issue.

In any case, our finding of more pronounced differences in HCs vs. mild AD participants than between aMCI vs. PD-MCI patients is similar to the trend we observed when using traditional neuropsychological screening tests (i.e., MMSE and MoCA), which give a dichotomous index of global cognitive functioning, indicating the presence/absence of cognitive impairment. In addition, the same pattern was found when considering specific neuropsychological tests. In a number of previous studies on this topic, authors devised SG assessment tools for evaluating specific aspects of cognition. For instance, Serino et al. (2017) developed an innovative measure for evaluating executive functions in cognitively normal PD, and Plancher et al. (2012) a test for assessing episodic memory in aMCI and AD, to mention just two. The SG devised in the present study aimed to provide an index of global functioning based on participant performance of several tasks, rather than on single aspects of cognition; the idea was to create a brief screening tool able to assess global cognitive functioning, as traditional neuropsychological screening tests do, but in ecologically relevant and standardized conditions (Rizzo et al., 2004; Saposnik and Levin, 2011). Therefore, the very fact that Smart Aging gave findings similar to those produced by conventional tools argues in favor of its use, as do the important advantages of SG-based assessment tools over traditional approaches. The fact that SGs are more user friendly, ecological, and motivating, as well as less time and resource consuming for the professionals involved are just some of these advantages (Bohil et al., 2011).

In previous research (Bottiroli et al., 2017), we have already shown that the five Smart Aging tasks pertain to different cognitive functions and engage the multi-domain skills involved in performing many real-life activities (Fortin et al., 2003). In particular, we showed that Smart Aging can be easily administered to evaluate memory, executive mechanisms, and visual–spatial processes, i.e., the abilities mainly supporting instrumental activities of daily living (Schmitter-Edgecombe et al., 2009). Hence, SGs like the Smart Aging platform, being devised as assessment tools, have added strengths, namely, they make it possible to assess how cognitive functions act together, as a whole, in a more ecological manner (Logan and Barber, 1985), and they used automated scoring systems (Clauser et al., 2002), which have several benefits for both patients and clinicians.

In Bottiroli et al. (2017), we considered cognitive functioning patterns across the five Smart Aging tasks, analyzing them in comparison with MoCA scores. In the present study, we decided to focus on accuracy, time, and distance across the tasks (i.e., to calculate and consider global accuracy, time and distance scores) as opposed to within each of them singly. There are two main reasons for this. As we already demonstrated (Bottiroli et al., 2017), it is not possible to separate the specific cognitive domains involved in performing individual tasks; instead, it is necessary to consider them acting as a whole, as they do during everyday life activities (Logan and Barber, 1985). In line with this, we indeed found the Smart Aging indices (global and total scores) to show significant correlations not only with MMSE/MoCA but also with all the specific neuropsychological tests considered. To further corroborate this point, it should be noted that considering each index within each single task would not have allowed us to capture the ecological added value of these platforms. In fact, researchers in the SG field usually consider performances in terms of global indices and not task by task (e.g., Raspelli et al., 2011; Cipresso et al., 2014; Ouellet et al., 2018). Second, we believe that each of the analyzed indices provides different information on participant performance. Accuracy is an index usually considered by traditional neuropsychological assessments, such as MMSE and MoCA, whereas time is usually considered in tests measuring attentional control, such as the Trail Making Test (Tombaugh, 2004). SG-based tools like Smart Aging offer additional indices, i.e., the distance covered while performing each activity in the virtual scenario, which may provide deeper insights on how individuals are able to effectively respond to environmental demands. According to the “stealth” approach (Shute et al., 2016), SGs are unique in that they allow performance to be measured by unobtrusively logging user behaviors, such as paths taken to reach destinations. In this context, the mild AD patients showed marked differences, compared with the other groups, in not only accuracy but also distance. The fact that the mild AD patients navigated the virtual scenario differently compared with HCs, and aMCI and PD-MCI patients may indicate that they were less able to be strategic and focused in responding to the task demands. Therefore, these features further support the view that SG assessment tools could provide a context for assessing a broader range of skills and constructs compared with traditional assessment approaches. Similarly, Cipresso et al. (2014) aimed to detect early executive function deficits in PD by considering indices such as task failure, time, strategies, and rule breaks during a VR-based test. Manera et al. (2015) on the other hand, considered time spent playing and number of errors in MCI and AD. Lee et al. (2014) devised the Virtual Radial Arm Maze in order to assess spatial working memory in aMCI and AD patients; they considered the number of times subjects reenter the same arm, the total time spent in the maze, and the total distance covered. Future studies should further explore the opportunities offered by the possibility of logging user behaviors in SG assessment tools. We suggest that the Smart Aging total score already represents a valuable parameter for evaluating individuals' global performances, given that it is based on simultaneous logging of user behaviors in terms of accuracy, time, and distance. After all, it could be that a subject obtains a high score in terms of accuracy, but takes a considerable amount of time, or does not cover an adequate distance within the virtual scenario, both findings that may reflect difficulties in strategic planning of responses to the demands. The Smart Aging total score efficiently discriminated not only HCs from mild AD patients but also aMCI patients from all the other groups, as shown by the ROC analyses. As a consequence, this index could be the one that best reflects participants' global cognition. Larger samples including individuals with/without cognitive impairment in early neurodegenerative disease, and with different types and levels of cognitive impairment, will allow more in-depth exploration of how each of the other indices—accuracy, time, and distance—may reflect different aspects of cognition in this population.

To date, the Smart Aging platform has been validated in a healthy population of older adults (Bottiroli et al., 2017). Cabinio et al. (2020) also tested it in aMCI patients compared with HCs and found significant differences between groups in all the indices considered (i.e., accuracy, time, and Smart Aging total score). In the present study, we confirmed and further extended those findings by also considering early AD and PD-MCI patients. To the best of our knowledge, this is the first study using an SG-based screening tool devised for assessing cognitive functioning in patients with different types and levels of cognitive impairment. Future studies are necessary to evaluate the performance of the Smart Aging platform in the screening of other neurodegenerative conditions. Another future challenge is to develop other scenarios and tasks with different levels of complexity, with a view to using this platform for remote monitoring of patient functioning and for rehabilitation purposes. For instance, this platform could be integrated into portable devices, such as tablets or laptops, and easily administered at patients' own homes. In recent years there has been a growing interest in telemedicine and telerehabilitation as means of providing rehabilitation remotely in chronic conditions, including ones related to aging, such as dementia and other neurodegenerative disorders (Nesbitt et al., 2000; Chirra et al., 2019). In this field, VR and SGs could allow remote delivery of different rehabilitation services in different medical conditions, benefiting patients and also healthcare systems in terms of cost effectiveness and feasibility for large-scale implementations (Zampolini et al., 2008; Peretti et al., 2017).

While we believe the findings we have reported are valuable and interesting, several limitations of the study suggest that they should be interpreted with caution. First, the number of participants (*n* = 91) may limit the generalizability of the results. In particular, the small sample size may explain why we were able to detect differences when they were marked, as in healthy controls and early AD patients, but not when they were more subtle, as when comparing amnesic and executive deficits in different types of MCI. This is, unfortunately, a limitation common to many studies conducted in clinical populations in this field (e.g., Cipresso et al., 2014; Lee et al., 2014; Manera et al., 2015; Tarnanas et al., 2015; Serino et al., 2017; Valladares-Rodriguez et al., 2018, 2019). Hence, a larger validation study should be performed. Second, the sample selection may constitute a further limitation of the present study. Our main aim was to differentiate between persons with different levels and types of cognitive impairment. To this end, we included patients at different points on the AD cognitive spectrum (i.e., mild AD, aMCI). Unfortunately, we did not cover the same range for the PD spectrum, as we included no Parkinson's disease with mild dementia patients. In addition, it would also be useful to consider patients showing comparable levels of global cognitive impairment, but the involvement of different cognitive domains (e.g., single-domain MCI vs. multiple-domain MCI) in order to further test the accuracy of the Smart Aging platform in identifying different types of early cognitive impairment. Third, in order to fully evaluate the full potential of Smart Aging as a screening tool for cognitive functioning, future studies are needed to assess its test–retest reliability and validity. The present study, however, provides initial evidence that an ecological evaluation of cognitive functioning performed with an SG-based assessment tool may offer a means of determining the presence/absence of cognitive impairment in neurodegenerative diseases.

Our study provides useful evidence that SG-based assessment tools may have a role to play in neuropsychological evaluation in the future. In particular, it suggests that the Smart Aging platform is a powerful screening tool for detecting the presence of cognitive deterioration. The many advantages offered by VR environments over traditional cognitive screening tests make this platform an innovative tool for clinicians and researchers interested in exploring cognitive mechanisms. We are now seeing a surge of interest in remote communication technologies as assessment tools (e.g., Geddes et al., 2020; Phillips et al., 2020; Scuteri et al., 2020) and treatment (Zucchella et al., 2018; Bloem et al., 2020; Maggio et al., 2020; Mantovani et al., 2020; Platz and Sandrini, 2020; Stasolla et al., 2020; Bernini et al., 2021) for use in all situations in which it is not possible to guarantee patients' continuity of care. In the context of the ongoing public health emergency, Smart Aging might be considered an innovative approach and valid support, making it possible to monitor cognitive function of individuals with neurodegenerative diseases remotely and safely in their own homes.

## Data Availability Statement

The datasets presented in this study can be found in online repositories. The names of the repository/repositories and accession number(s) can be found at: Zenodo. http://doi.org/10.5281/zenodo.4422021.

## Ethics Statement

The studies involving human participants were reviewed and approved by Ethical Committee of the San Matteo Hospital in Pavia. The patients/participants provided their written informed consent to participate in this study.

## Author Contributions

SBo designed the game, collected the data, carried out the statistical analyses, and wrote the manuscript. SBe assisted in collecting the data, statistical analyses, interpretation of the results, and manuscript writing. CZ and EC designed the game and assisted with the selection of the clinical assessments used in the study. SP and PC supervised the data collection and storage, and assisted in data analyses. DT developed the game. TV, ES, CT, and GS supervised the entire study. All authors did read and approve the final version of the manuscript.

## Conflict of Interest

The authors declare that the research was conducted in the absence of any commercial or financial relationships that could be construed as a potential conflict of interest.
